# PD-L1 Expression Correlated with Clinicopathological Factors and Akt/Stat3 Pathway in Oral SCC

**DOI:** 10.3390/life12020238

**Published:** 2022-02-04

**Authors:** Dong-Ho Geum, Dae-Seok Hwang, Chang-Hun Lee, Sung-Dae Cho, Min-A Jang, Mi-Heon Ryu, Uk-Kyu Kim

**Affiliations:** 1 Department of Oral and Maxillofacial Surgery, School of Dentistry, Pusan National University, 49, Busandaehak-ro, Mulgeum-eup, Yangsan 50612, Korea; gdh84@daum.net (D.-H.G.); dshwang@pusan.ac.kr (D.-S.H.); 2 Department of Pathology, School of Medicine, Pusan National University, 49, Busandaehak-ro, Mulgeum-eup, Yangsan 50612, Korea; cnlee@pusan.ac.kr; 3 Department of Oral Pathology, School of Dentistry and Dental Research Institute, Seoul National University, Seoul 03080, Korea; efiwdsc@snu.ac.kr; 4 Dental and Life Science Institute, Pusan National University, 49, Busandaehak-ro, Mulgeum-eup, Yangsan 50612, Korea; luxury8692@naver.com; 5 Department of Oral Pathology, Dental and Life Science Institute, School of Dentistry, Pusan National University, 49, Busandaehak-ro, Mulgeum-eup, Yangsan 50612, Korea

**Keywords:** programmed cell death ligand 1, oral squamous cell carcinoma, prognosis, immunotherapy

## Abstract

Programmed cell death ligand 1 (PD-L1) is an immune checkpoint molecule that inhibits immune responses. The physiological and prognostic role of the PD-L1 signaling pathway in the oral maxillofacial region is unclear. This study aimed to investigate the role of PD-L1 in the progression of oral squamous cell carcinoma (OSCC). Furthermore, clinicopathological factors related to PD-L1 expression were examined in patients with OSCC through immunohistochemistry (IHC) of tissue sections and through an in vitro study in OSCC cells. The medical records, radiographic findings, and mortality referrals of 81 patients obtained from the National Statistical Office were reviewed. IHC was performed on tissue specimens of these patients to determine the expression levels of PD-L1, which showed significant statistical differences based on age, tumor size, TNM stage, cervical lymph node metastasis, and locoregional recurrence. Patients with a high PD-L1 expression had significantly poorer survival rates. Multivariate analysis using the Cox proportional model confirmed the high relative risk ratio for high PD-L1 expression, TNM stage, and neck node metastasis, all of which were significantly associated with a poor prognosis in patients with OSCC. The in vitro study showed that SAS and YD38 cells transfected with PD-L1 siRNA had significantly increased apoptosis, reduced proliferative capacity, and tumorigenicity.

## 1. Introduction

Oral squamous cell carcinoma (OSCC) is an immunosuppressive disease that inhibits the normal host immune system through various mechanisms, including altered immunogenicity against host immune cells or expressed immunosuppressive mediators [1]. Immune checkpoint-based programmed cell death 1 receptor (PD-1) and its ligand, programmed cell death ligand 1 (PD-L1), are widely known to play a role in tumors and, recently, targeted therapies that block the receptor–ligand interaction have been approved for various malignant diseases [2].

PD-1 is an immune checkpoint protein expressed on the surface of immune cells, such as cytotoxic T cells [3,4,5]. T cells cannot provide a cytotoxic immune response when PD-L1 and PD-L2, which are overexpressed on the surface of cancer cells, bind to the transmembrane protein PD-1 on T cells [6,7,8].

Anti-PD-L1 and anti-PD-L2 cancer immunotherapy drugs, which target the immune evasion mechanism used by cancer cells, have been recently developed. These drugs have antagonist effects by blocking the binding of PD-L1, which is expressed on cancer cells, to PD-1 receptors on T cells, thereby maintaining the cytotoxic activity of T cells [9,10,11]. A recent report has shown limited, but positive outcomes in a study of patients with recurrent or metastatic head and neck SCC who were administered pembrolizumab [11]. In addition, Ferris et al. reported that the administration of nivolumab was associated with increased survival in patients with recurrent or metastatic head and neck SCC who had initially received cetuximab [12].

The role of PD-L1 in oral cancer is not yet fully understood. We examined various biomarkers that are expressed in OSCC tissues from previous studies, and selected PD-L1 for this study. We analyzed the prognosis of patients with OSCC who underwent surgery at Pusan National University Dental Hospital and examined their clinicopathological data. Tumor specimens, which were intraoperatively extracted from patients, were subjected to immunohistochemistry (IHC) analysis for determining the correlation between the PD-L1 expression and impact factors. Furthermore, we performed in vitro experiments and elucidated the oncogenic mechanism of PD-L1 to identify the association between OSCC and PD-L1. These findings can be used to predict the prognosis of patients with cancer, as well as to develop treatment strategies for better patient outcomes.

## 2. Materials and Methods

### 2.1. Analysis of Clinical Data of Patients

We selected 541 patients who were diagnosed with OSCC and underwent surgery for OSCC between January 2000 and December 2013 at Pusan National University Hospital, and oral and maxillofacial surgery at Pusan National University Dental Hospital. The medical records of patients who were treated in the same department were retrospectively analyzed. Subsequently, with the cooperation of the National Statistical Office, factors affecting long-term survival rate were analyzed by examining those responsible for patient death and the date of death. Among the 541 patients diagnosed with OSCC through histopathological examination, the final number of subjects was 81, all of which were selected using the following inclusion criteria:

- Patients confirmed to have OSCC through biopsy in this department;
- Patients treated with radical resection in this department;
- Patients not treated for their primary tumor site at another hospital before surgery;
- Patients with no history of malignancy in other parts of the body;
- Patients without loss or contamination of tissue specimens.

Among the 81 patients selected as the final study subjects, we examined patient gender, age, drinking status, history of smoking, primary site, histodifferentiation, clinical stage, postoperative locoregional recurrence, and cervical nodal metastasis. For smoking history and drinking status, those who smoked less than 0.5 cigarettes per day and drank less than 1 glass of beer per day were disregarded. The TNM clinical stages of patients were classified according to the TNM staging classification for the lip and oral cavity from the eighth edition of the American Joint Committee on Cancer (2016). Following this, we analyzed the survival status of patients or the date and cause of death using the data provided by the National Statistics Office. This study was conducted in accordance with the guidelines of the Declaration of Helsinki and was approved by the Institutional Review Board (IRB) of Pusan National University Hospital and Pusan National and University Dental Hospital (IRB No. H-1808-017-070, PNUDH-2017-004).

### 2.2. Tissue Specimens

Paraffin-embedded blocks containing OSCC tissue samples were prepared using specimens extracted intraoperatively from patients included in this study. Histopathological evaluation was performed on hematoxylin−eosin stained slides, and the tumor type and level of malignancy were determined following the guidelines of the World Health Organization classification for head and neck tumors. Prior to the treatment of the tissue specimen, histopathological analysis was conducted on surgical tissue specimens using the avidin−biotin complex method.

### 2.3. Analysis of Gene Expression Profile Using UALCAN

UALCAN (http://ualcan.path.uab.edu, accessed on 30 June 2021) is a portal for analyzing the gene expression of tumors, using data extracted from the TCGA system. We assessed *PD-L1* profiling in various cancers using UALCAN and analyzed the mRNA expression of *PD-L1* in 23 tumors for further study.

### 2.4. Immunohistochemical Analysis

Unstained tissue sections were deparaffinized, treated with 100% alcohol, and washed with phosphate-buffered saline. For antigen retrieval, sections were boiled in a citrate buffer (pH 6.0) for 10 min using a hot plate and then cooled for 1 h at room temperature. Endogenous peroxidases were blocked with a peroxidase blocking solution (Sigma-Aldrich, Saint Louis, MO, USA) for 10 min, and sections were treated with a protein-blocking solution for 20 min. These were incubated with the primary antibody against PD-L1 (Abcam) overnight at 4 °C, and subsequently with the appropriate secondary antibody (SuperPictureTM third Gen IHC Detection kit) for 10 min at 37 °C. Sections were then stained with freshly prepared DAB substrate (Dako), counterstained with Mayer’s hematoxylin, dehydrated, mounted, and examined under a light microscope. To analyze the sections, five non-overlapping fields per slide were randomly selected and images were captured using a light microscope attached to a digital camera (Olympus, BX51T, Tokyo, Japan, 100×).

### 2.5. Evaluation of PD-L1 Expression

The percentage of the stained area was categorized as 0% (0 point), 1–25% (1 point), 26–50% (2 points), 50–75% (3 points), or 75% or higher (4 points). The staining intensity was categorized as negative (0 point), weak diffuse (1 point), moderate granular (2 points), or strong granular (3 points) after observing the nuclear, cytoplasmic, and cell membrane intensities. The final staining scores were calculated by multiplying the percentage of the stained area with the staining intensity. The mean value of the PD-L1 expression was obtained per slide for each patient, and the patients were divided into a group with a mean expression level higher than that of all patients, and a group with a lower mean expression level [13].

### 2.6. In Vitro Study

To understand the PD-L1 expression and its mechanism of action in cancer cells, the effect of the inhibition of PD-L1 expression was examined in OSCC cells following PD-L1 knockdown after transfection with an siRNA targeting PD-L1. Using Western blotting, we evaluated the cell viability, nuclear fragmentation, invasive ability, and protein expression. These parameters were also evaluated in the control cells transfected with a non-targeting siRNA.

### 2.7. Cell Culture

YD38 cells (OSCC cell line, a kind gift from Professor Jin Kim, Yonsei University College of Dentistry) and SAS cells (OSCC cell line, a kind gift from Professor Sung-Dae Cho, Seoul National University, School of Dentistry) were used in this study. These cells were cultured in Roswell Park Memorial Institute 1640 medium and Dulbecco’s modified Eagle medium/F-12 supplemented with 10% fetal bovine serum (FBS; Hyclone) and 1% penicillin/streptomycin (Invitrogen, New York, NY, USA). The cells were maintained in a humidified atmosphere at 37 °C with 5% CO_2_, with the culture medium changed once every three days.

### 2.8. siRNA Transfection

Two siRNAs targeting PD-L1 were purchased from Bioneer (Bioneer Inc., Daejeon, Korea). The siRNA sequences were as follows:

siPD-L1(hu)1 RNA 5′-CCU ACU GGC AUU UGC UGA ACG CAU U-3′ (1-AS),
siPD-L1(hu)2 RNA 5′-AAU GCG UUC AGC AAA UGC CAG UAG G-3′ (1-AA)

A non-targeting siRNA that did not target any known human genes was purchased from Dharmacon (Lafayette, CO, USA) and used as a negative control. siRNA transfection was performed using Oligofectamine (Invitrogen) according to the manufacturer’s instructions. RNA and proteins were extracted 48 h after transfection.

To understand the mode of action of PD-L1, the cells were treated with SC79 (Sigma-Aldrich Chemical Co., Saint Louis, MO, USA) or recombinant CXCL8 (Biotechne, Minneapolis, MN, USA) 2 h before transfection with PD-L1 siRNA. Subsequently, the cells were transfected with PD-L1 siRNA, protein extraction was performed, and the proteins were further analyzed.

### 2.9. Cell Viability Assay

The effects of siRNA transfection on cell viability were analyzed using the cell counting kit-8 (CCK-8) assay. The cells were stained with a CCK-8 solution (Dojindo Molecular Technologies Inc., Kumamoto, Japan) and the optical density was determined using a microplate reader.

### 2.10. Scratch Wound Healing Assay

The scratch wound healing assay was performed using SAS and YD38 cells. Briefly, the cells were seeded in a six-well culture plate and grown for 24 h after transfection with PD-L1 siRNA until they reached 70–80% confluency. Then, the cell monolayer was scratched using a sterile 1 mL pipet tip for a distance of 4 cm. After incubation for 24 h or 48 h, photographs were taken using a phase contrast microscope and the distance covered by the scratched wound edge was measured using Image J.

### 2.11. Hoechst 33,342 Staining

After transfecting SAS or YD38 cells with PD-L1 siRNA, they were seeded in a 12-well culture plate. After 24 h or 48 h, the cells were stained with 1 µg/mL Hoechst 33,342 (Thermo Fisher Scientific, Waltham, MA, USA), incubated at 37 °C for 15 min, and observed using a fluorescence microscope (λEx 350 nm/λEm 461 nm).

### 2.12. ANNEXIN V/PI Staining

PD-L1 siRNA-transfected SAS or YD38 cells were seeded in a six-well culture plate at a density of 1 × 10^6^ cells per well and they were incubated for 24 h at 37 °C. The cells were then resuspended in 1 mL of 1x binding buffer. Subsequently, 100 μL of this solution was transferred into a 5 mL culture tube. Next, 5 µL of Annexin V (BD Biosciences, San Jose, CA, USA) followed by 5 µL of propidium iodide was added. The cells were incubated for 15 min in the dark and then 1× binding buffer was added. These were later analyzed by flow cytometry.

### 2.13. Colony-Forming Assay

After 48 h of transfection, the cells were trypsinized and resuspended in medium supplemented with 0.3% soft agar (5 × 10^1^, 1 × 10^2^, 2 × 10^2^), and placed on 0.5% agarose. After 14 days, the colonies formed were counted.

### 2.14. Invasion Assay

To compare the invasive ability between the cells transfected with siRNAs targeting PD-L1 and those transfected with a non-targeting siRNA, an invasion assay was performed using Matrigel-coated Transwell cell culture chambers (8 μm pore size; Corning Incorporated, Corning, NY, USA). The cells were cultured in a serum-free medium and resuspended. Subsequently, 40 μL of the cell suspension was coated onto the membrane of the Transwell plate and air-dried for 3 h. Next, 5 × 10^4^ cells were added to the serum-free medium and placed in the upper chamber. The medium (600 μL) containing 10% FBS was added in the lower chamber, and plates were incubated at 37 °C in a 5% CO_2_ atmosphere for 24 h and 48 h. Cells that did not penetrate the membrane in the upper chamber were removed using a cotton swab. Those on the lower surface that passed through the Matrigel and membrane were fixed with 4% formaldehyde and later stained with 2% crystal violet in 2% ethanol. Cells were imaged and counted under a light microscope at 200× magnification using three randomly selected sections from each membrane.

### 2.15. Western Blot Analysis

Cell lysates were prepared using a lysis buffer and the protein concentration of each sample was measured using the DC protein analysis kit (BIO-RAD Laboratories). After normalization, lysates containing the same amount of protein were separated by sodium dodecyl sulfate polyacrylamide gel electrophoresis (SDS-PAGE) and the separated proteins were transferred onto an Immuno-Blot PVDF membrane. The membrane was blocked with 5% skimmed milk in Tris-buffered saline containing Tween-20 (TBST) for 2 h at room temperature and then incubated with the primary antibody, followed by the appropriate secondary antibody conjugated with horseradish peroxidase. Antibodies against cleaved poly ADP ribose polymerase (PARP), PD-L1, signal transducer and activator of transcription (Stat) 3, phosphorylated Stat 3, chemokine (C-X-C motif) ligand (CXCL) 8, Akt, phosphorylated Akt, ERK, and phosphorylated ERK were purchased from Cell Signaling Technology (Danvers, MA, USA). The anti-actin antibody was obtained from Santa Cruz Biotechnology. Immunoreactive bands were visualized using ImageQuant LAS 500 imager (GE Healthcare Life Sciences).

### 2.16. Statistical Analysis

We used the χ^2^ test to determine the clinical factors that showed a significant difference based on the PD-L1 expression levels. Spearman correlation analysis was used to determine the correlation between the PD-L1 expression levels and clinical factors and their correlation coefficients. Student’s *t*-test was performed to analyze the distribution and to compare the differences in PD-L1 expression levels between the normal oral mucosa (NOM) group and OSCC patient group, as assessed by IHC. The survival rate of patients with OSCC was schematized using the Kaplan–Meier method, and the log-rank test was used to compare the PD-L1 expression levels and the survival rate for each factor. Using the Cox proportional hazards model, the relative risks of locoregional recurrence, cervical nodal metastasis, and PD-L1 expression were assessed to determine their usefulness as oral cancer biomarkers. All statistical analyses were performed using SPSS software (version 21; SPSS, Inc., Chicago, IL, USA). In the in vitro study, all variables were tested in three independent experiments, and the data are represented as mean ± SD. Results with a *p* value of <0.05 indicated statistical significance.

## 3. Results

### 3.1. Overview of the Clinical Data

Table 1 summarizes the overall clinicopathological factors of the 81 patients included in this study. The TNM classification from the preoperative diagnosis was categorized as the clinical TNM (cTNM), and the final TNM classification reflecting postoperative histopathological analysis was categorized as pathologic TNM (pTNM). From the pTNM group that underwent histopathological evaluation, patients were reclassified based on tumor size: T1 and T2 were categorized as low grade and T3 and T4 as high grade.

TNM classification from the preoperative diagnosis was categorized as cTNM, and the final TNM classification reflecting the postoperative histopathological analysis was categorized as pTNM. From the pTNM group that underwent histopathological evaluation, the patients were re-classified according to the size of their tumors: T1 and T2 were categorized as low grade (45 patients) and T3 and T4 as high grade (36 patients).

Thirty patients (36.8%) showed cervical nodal metastasis during the study period and 28 patients (35.3%) showed loco-regional recurrence. Eight patients (9.8%) were found to have distant metastasis. Among those with distant metastasis, four cases had no or identified cervical nodal metastasis.

A histo-differentiation analysis showed that 49 patients had well-differentiated tumors (57.7%), 28 patients had moderately-differentiated tumors (36.6%), and 4 patients had poorly-differentiated tumors (5.6%). Mixed differentiation was considered to be a less differentiated state (Table 1).

### 3.2. Analysis of PD-L1 Expression Using UALCAN Portal

**Figure 1:**
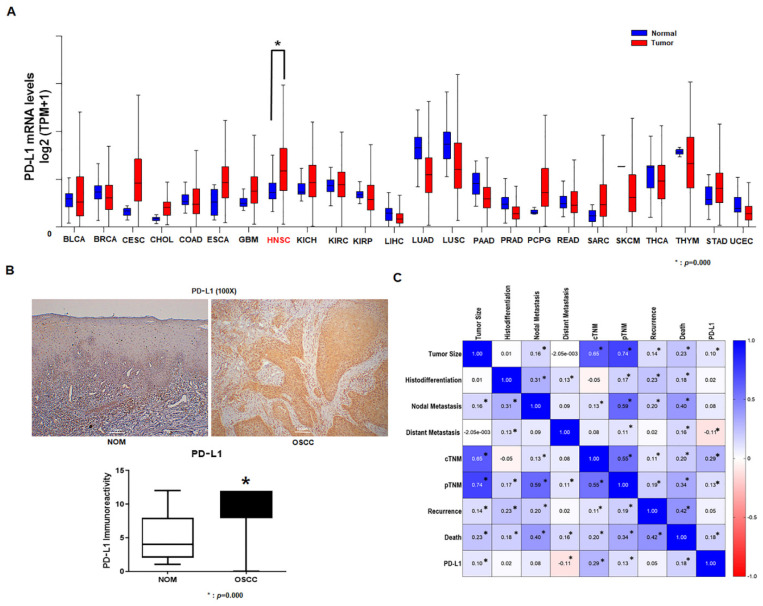
Upregulation of PD-L1 in oral cancer and its clinicopathological correlation. Assessment of *PD-L1* mRNA expression between 23 normal human tissues and 23 tumor tissues using data from the UALCAN portal (**A**) (* *p* = 0.000). Immunohistochemical staining of PD-L1 expression in NOM and cancer tissue from patients with OSCC. High levels of PD-L1 expression can be seen in the cytoplasm and cell membrane of tumor cells. Student’s *t* test was performed to assess the levels of PD-L1 immunoreactivity in NOM and in cancer tissue from patients with OSCC. PD-L1 expression in cancer tissue from OSCC patients is significantly higher than in the mucosal tissue from the NOM group (original magnification 100×) (**B**). Spearman correlation coefficient was determined based on PD-L1 expression and clinicopathological factors of OSCC patients. Factors such as tumor size, distant metastasis, cTNM, pTNM, and death of OSCC patients showed a correlation with PD-L1 expression (**C**) (* *p* < 0.05). NOM, normal oral mucosa; OSCC, oral squamous cell carcinoma; IHC, immuno-histochemistry.

Using UALCAN that uses TCGA data, we investigated the PD-L1 mRNA expression in 23 normal human tissues and corresponding tumor tissues. Our results revealed that PD-L1 mRNA (Figure 1A) was clearly increased in the human head and neck cancer tissues compared to normal tissues.

### 3.3. Expression of PD-L1 in Tumor Specimens

The mean staining scores were obtained from five tissue slides per patient from the NOM group and from the OSCC patient group, and were used as a representative score for each patient. Patients who had representative scores higher than the overall mean score of 8.17 were categorized as the PD-L1 high-expression group, whereas those with representative scores lower than 8.17 were categorized as the PD-L1 low-expression group. The mean value of PD-L1 expression in 12 patients from the NOM group was 5.28, and the SD was 3.351. The mean PD-L1 expression level in 81 tissues from patients with OSCC was 8.17, and the SD was 3.473. Student’s *t*-test analysis revealed a significant difference between the OSCC patient group and the control group (Figure 1B). In addition, the results of the Spearman correlation analysis revealed that the PDL-1 expression in OSCC patients was significantly correlated with tumor size, distant metastasis, cTNM, pTNM, and death (Figure 1C).

### 3.4. Analysis of Survival Rate

Among the 81 patients, 29 (35.5%) survived until the monitoring date (1 October 2019), and 35 (43.2%) died from oral cancer. Furthermore, 17 patients died from a cause unassociated with oral cancer (20.3%). The mean follow-up period for all patients was 76.0 ± 69.4 months, showing a wide range from 1 to 266 months. To estimate prognosis, the survival time for patients who died was set from the date of their first major operation until the date of their death, and patients who were alive after follow-up was completed (1 October 2019) were considered as surviving patients. The survival period of patients who died from a cause other than cancer used censored data.

The overall five-year survival rate for all patients, obtained from the Kaplan–Meier survival analysis, was 60.1%, and the 10-year survival rate was 54.8%. The survival curve displays differences in survival rate based on each clinical factor and PD-L1 expression levels, as assessed by the IHC analysis.

**Figure 2:**
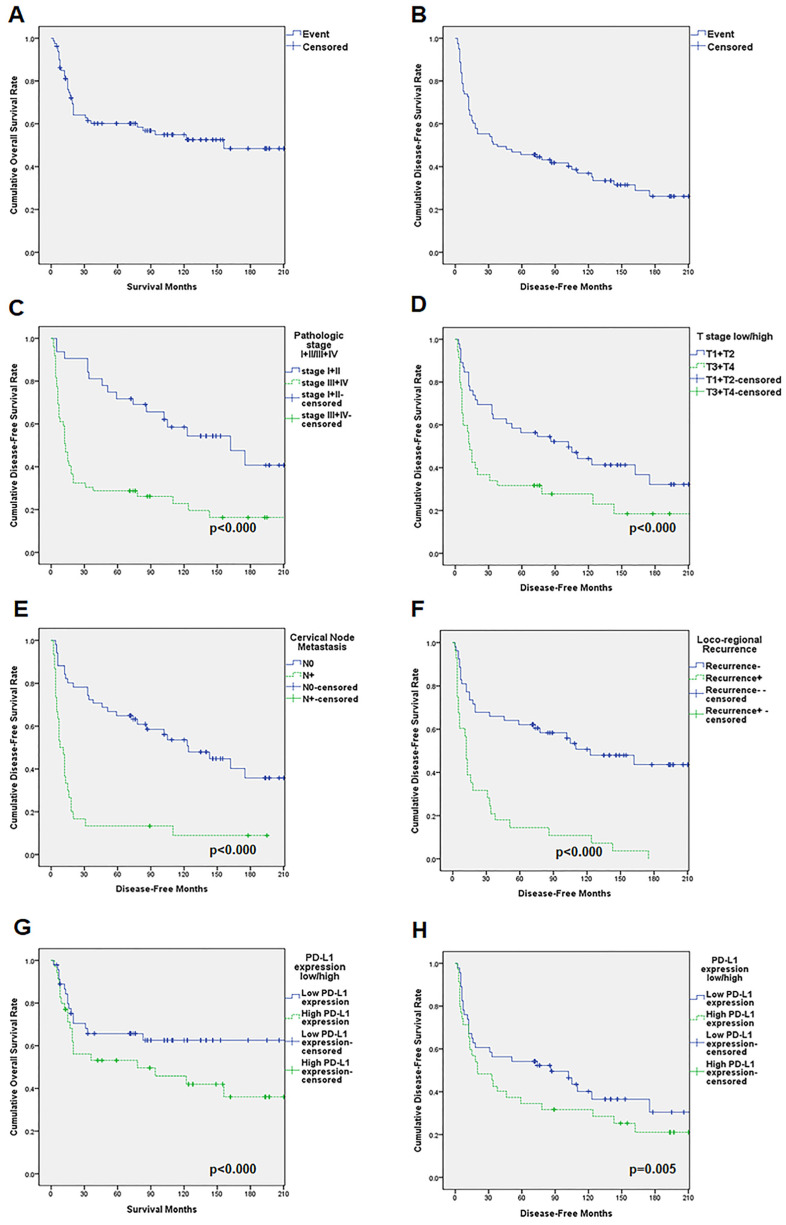
Cumulative survival rates for patients with OSCC as assessed by the Kaplan–Meier method. The overall five-year survival rate was 60.1%, and the 10-year survival rate was 52.5% (**A**). For disease-free survival, the 5-year survival rate was 45.8% and the 10-year survival rate was 37.0% (**B**)**.** Patients with late-stage disease (stage III and IV) showed significantly poorer survival rates (*p* < 0.000) (**C**). For stage I + II patients, the disease-free 5-year survival rate was 71.7% and the 10-year survival rate was 58.5%. For stage III + IV patients, the disease-free 5-year survival rate was 28.7% and the 10-year survival rate was 22.8%. Patients with large tumor sizes had significantly poorer survival rates (*p* < 0.000). For T1 + T2 patients, the disease-free 5-year survival rate was 56.3% and the 10-year survival rate was 44.3%. For T3 + T4 patients, the disease-free 5-year survival rate was 31.6% and the 10-year survival rate was 27.7% (**D**). Patients with cervical node metastasis showed significantly poorer survival rates (*p* < 0.000). In N0 patients, the disease-free 5-year survival rate was 64.8% and the 10-year survival rate was 60.9%. In N+ patients, the disease-free 5-year survival rate was 13.3% and the 10-year survival rate was 8.9% (**E**). Patients with locoregional recurrence had a significantly lower survival rate than patients with a negative recurrence (*p* < 0.000). For patients who did not have locoregional recurrence, the disease-free 5-year survival rate was 62.1% and the 10-year survival rate was 50.7%. For patients with locoregional recurrence, the disease-free 5-year survival rate was 14.4% and the 10-year survival rate was 10.8% (**F**). Overall survival rate based on PD-L1 expression levels. Patients with high PD-L1 expression levels had a 5-year survival rate of 53.1% and a 10-year survival rate of 42.0%. Patients with low PD-L1 expression levels had a 5-year survival rate of 65.7% and a 10-year survival rate of 62.6% (*p* < 0.000) (**G**). Patients with low levels of PD-L1 expression had a disease-free 5-year survival rate of 54.1% and a disease-free 10-year sur-vival rate of 40.1%. Patients with high levels of PD-L1 expression had a disease-free 5-year survival rate of 34.5% and a disease-free 10-year survival rate of 31.6% (*p* = 0.005) (**H**). OSCC, oral squamous cell carcinoma.

In patients with low PD-L1 expression levels, the five-year survival rate was 78.2% and the 10-year survival rate was 65.4%, whereas in patients with high PD-L1 expression levels, the 5-year and 10-year survival rates were 53.7% and 51.3%, respectively (Figure 2).

### 3.5. Association of PD-L1 Expression Levels with Clinicopathological Factors

Patients with OSCC were divided into high and low expression groups based on their mean PD-L1 expression values, and their association with clinicopathological factors was analyzed using the χ^2^ test. The percentage of patients with a high PD-L1 expression in the buccal mucosa was relatively high. Patient age, Tumor size, cTNM, pTNM, Histopathological grade, Cervical nodal metastasis, and Locoregional recurrence demonstrated statistically significant differences. Notably, PD-L1 expression levels were very strongly positively correlated with tumor size and factors indicating cancer progression, such as cTNM and pTNM. Their association with Cervical nodal metastasis, which could be considered as a dependent variable, was also shown to be significant. In contrast, no significant differences were observed with respect to Gender, Drinking, Smoking, or Distant metastasis (Table 1).

The factors shown in previous studies that were correlated with the rate of patient survival and PD-L1 expression levels were examined using a multivariate analysis with the Cox proportional hazard model, and the relative risk and significance probability were determined. Along with pTNM (1.419), cervical nodal metastasis (3.053) and locoregional recurrence (1.837), which have been shown by previous studies to be risk factors, as well as PD-L1 expression levels (1.573) also indicated a relative risk significantly higher than 1. In addition to the above factors and the higher than average PD-L1 expression levels, the risk of mortality significantly increased (Table 2).

### 3.6. In Vitro Study

**Figure 3:**
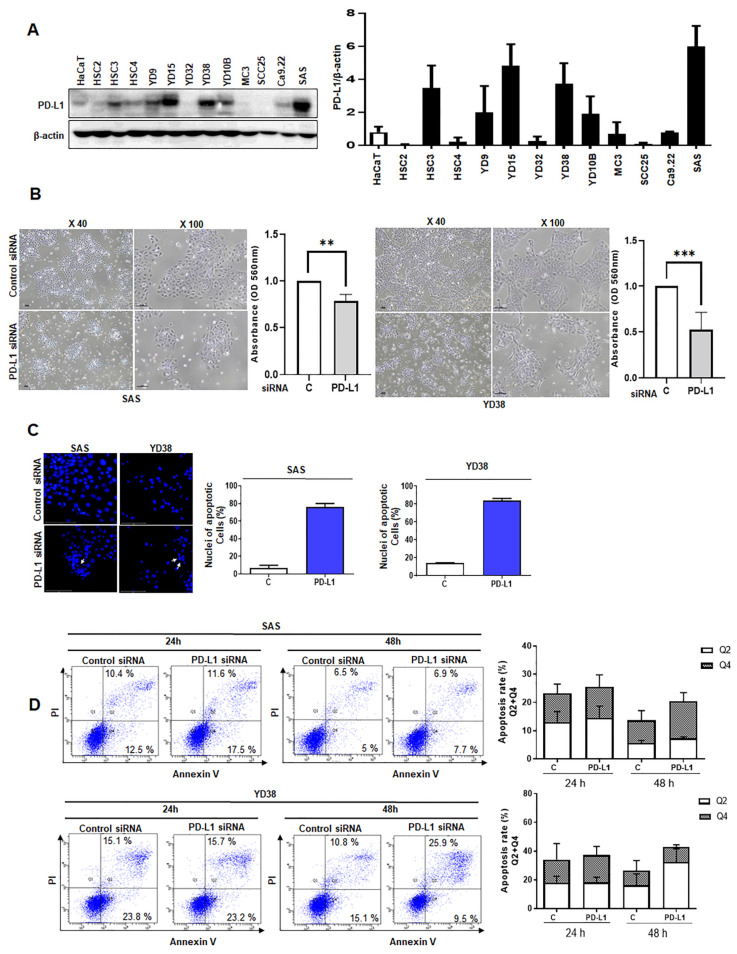
The decrease in cell viability and the assessment of apoptosis in SAS and YD38 cells. The expression levels of PD-L1 in HaCaT cells and various OSCC cell lines were determined using West-ern blotting (**A**). Differences in the proliferative capacity between PD-L1 siRNA-transfected SAS and YD38 cells and non-targeting siRNA-transfected SAS and YD38 cells were observed after 48 h of transfection, with the non-targeting siRNA-transfected SAS and YD38 cells being more prolif-erative than the PD-L1 siRNA-transfected cells (original western blotting figures see Supplementary Materials) (**B**). Nuclear fragmentation assay in OSCC cells transfected with PD-L1 siRNA. Nuclear fragmentation including apoptotic bodies can be observed in PD-L1 siRNA-transfected YD38 cells by immunofluorescence analysis (white arrow). The nuclear fragmentation rate was significantly increased in both cell lines (**C**). PD-L1 siRNA-transfected SAS and YD38 cells showed an increase in early apoptosis (Q2) and late apoptosis (Q4) at 24 h and 48 h after PD-L1 siRNA transfection (**D**). These data indicate that the rate of apoptosis was significantly increased in both the cell lines upon siRNA-mediated knockdown of PD-L1. The results are shown as the mean value ± SD (*n* = 3), and were analyzed by Student’s *t* test (** *p* < 0.01, *** *p* < 0.001). Original magnification 400x. OSCC, oral squamous cell carcinoma; SD, standard deviation.

Prior to the in vitro study, Western blotting was performed to examine the levels of PD-L1 protein expression in HaCaT cells and various OSCC cell lines. Consequently, high levels of PD-L1 protein expression were observed in SAS and YD38 cells, and these cell lines were used for further study (Figure 3A).

In the cell viability assay, the cell number decreased in both cell lines due to the knockdown of PD-L1 by siRNA, with the YD38 cell line showing a more significant decline in cell viability than that in the SAS cell line (Figure 3B).

We performed an immunofluorescence analysis of the cells transfected with PD-L1 siRNA or non-targeting siRNA to examine the changes in the nuclear shape and to assess the presence of apoptotic bodies. Increased nuclear shrinkage was observed in cancer cells following PD-L1 knockdown. In addition, the fragmentation rate was significantly increased and apoptotic bodies were observed, suggesting that PD-L1 knockdown increased apoptosis (Figure 3C).

The results of ANNEXIN V/PI staining revealed that, compared to SAS and YD38 cells transfected with non-targeting siRNA, PD-L1-siRNA transfected SAS and YD38 cells showed an increase in early apoptosis and late apoptosis at 24 h and 48 h after PD-L1 siRNA transfection (Figure 3D).

### 3.7. Analysis of Tumorigenic Capacity

**Figure 4:**
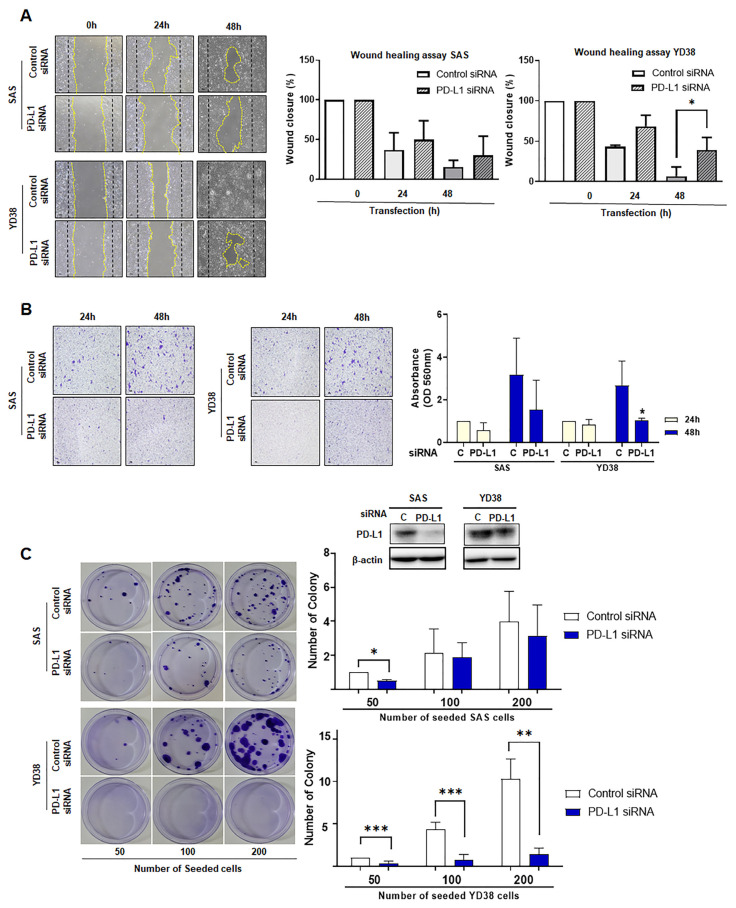
Differences in the scratch wound healing assay, invasive, and colony forming capacity between non-targeting siRNA-transfected cells and cells transfected with PD-L1 siRNA were ob-served after 24 h and 48 h of transfection. Cells transfected with non-targeting siRNA were more capable of healing wounds (**A**) and being invasive (**B**) than PD-L1 siRNA-transfected cells. In addi-tion, the ability of colony forming of the cells transfected with non-targeting siRNA was higher than PD-L1 siRNA-transfected cells (**C**) (* *p* < 0.05, ** *p* < 0.01, *** *p* < 0.001). (original western blotting figures see Supplementary Materials).

In the scratch wound healing assay, SAS and YD38 cells transfected with PD-L1 siRNA revealed a decrease in closure of the wound area, as compared to cells transfected with the non-targeting siRNA after 24 h or 48 h of incubation (Figure 4A).

In the invasion assay, cells transfected with PD-L1 siRNA showed a significant decrease of cells in cell staining area as compared to cells transfected with the non-targeting siRNA after 48 h of incubation. Although both SAS and YD38 cells with PD-L1 knockdown showed a greater difference compared to cells transfected with non-targeting siRNA, YD38 cells showed significantly decreased invasiveness after PD-L1 knockdown (Figure 4B).

In the colony-forming assay using SAS and YD38 cells, after two weeks of incubation, cells treated with non-targeting siRNA were significantly increased in number compared to cells with PD-L1 knockdown. After two weeks of incubation, we observed a statistically significant difference between YD38 cells transfected with PD-L1 siRNA and those transfected with non-targeting siRNA, although the difference was smaller than that observed in SAS cells (Figure 4C).

The colony-forming assay was performed to evaluate the effect of siRNA-mediated knockdown of PD-L1 on the tumorigenesis of SAS and YD38 cells. After 2 weeks of incubation, PD-L1 knockdown reduced the anchorage independent growth of SAS and YD38 cells compared to control cells transfected with non-targeting siRNA. SAS and YD38 cells transfected with siRNA targeting PD-L1 showed significantly reduced proliferation. The results are shown as the mean value ± SD (*n* = 3) and were analyzed by Student’s *t* test (* *p* < 0.05, ** *p* < 0.01, *** *p* < 0.001).

### 3.8. Western Blotting

**Figure 5:**
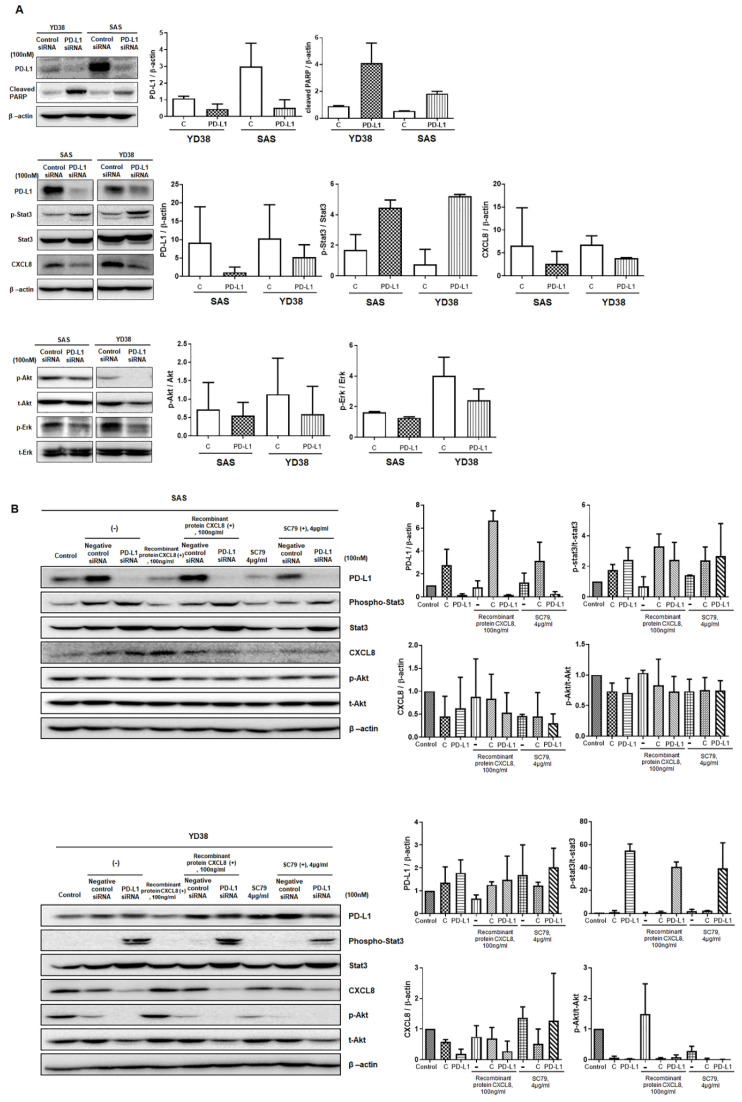
Effect of PD-L1 knockdown on the expression of apoptosis-related proteins and proteins related to the PD-L1 pathway in SAS and YD38 cells. β-actin was used as a loading control. Western blotting was performed to measure PD-L1, cleaved PARP, phosphorylated Stat3, Stat3, CXCL8, phosphorylated Akt, Akt, phosphorylated ERK, and ERK levels (**A**). The levels of PD-L1 were sig-nificantly decreased in YD38 and SAS cells transfected with PD-L1 siRNA. The levels of cleaved PARP and phosphorylated Stat3 were increased significantly, while those of CXCL8, phosphory-lated Akt, and phosphorylated ERK were decreased in YD38 and SAS cells transfected with PD-L1 siRNA (**A**). SC79 and CXCL8 pretreatment repressed the apoptosis of PD-L1 siRNA-transfected SAS and YD38 cells via Akt phosphorylation. SC79 pretreatment restored the expression levels of phos-phorylated Akt in SAS cells, while it diminished the expression of phosphorylated Stat3 (**B**). (original western blotting figures see Supplementary Materials).

In both SAS and YD38 cells, the siRNA-mediated knockdown of PD-L1 significantly decreased the expression levels of the PD-L1 protein, whereas the expression of cleaved PARP was significantly increased compared to that of the control group (Figure 5A). Furthermore, the levels of phosphorylated Stat3 were significantly increased, while those of CXCL8, phosphorylated Akt, and phosphorylated ERK were decreased in SAS and YD38 cells transfected with PD-L1 siRNA (Figure 5B). To understand the mode of action of PD-L1 expression, the cells were pretreated with SC79 or CXCL8 2 h before PD-L1 siRNA transfection. SC79 pretreatment restored the expression levels of phosphorylated Akt in SAS cells and affected the expression of phosphorylated Stat3, indicating a decrease in pStat3. In contrast, CXCL8 pretreatment did not affect the expression levels of phosphorylated Stat3, CXCL8, and phosphorylated Akt.

## 4. Discussion

Few studies have been published on the correlation of OSCC with PD-L1 expression, associated prognosis factors, and differences in PD-L1 expression levels based on tumor progression in the oral and maxillofacial areas. Moreover, the results of these previous studies have been inconsistent.

In our study, PD-L1 demonstrated a statistically significant strong positive correlation with the factors associated with tumor progression stage, such as tumor size, cTNM, and pTNM. Specifically, among patients with high PD-L1 expression levels, a 2.5-fold higher percentage of patients with pathologic stages III and IV were observed than those with pathologic stages I and II.

Additionally, we found that locoregional recurrence, cervical nodal metastasis, and PD-L1 expression were significantly correlated. Taken together, the PD-L1 expression was associated with tumor growth and cervical nodal metastasis, and demonstrated a tendency to increase at progressive stages. Regarding tumor growth and progression, Yagyuu et al. reported in their study of oral precancerous lesions that PD-L1/PD-1 expression in the epithelial and subepithelial areas was associated with the malignant transformation of precancerous lesions [14,15].

Our results also revealed a significant association between PD-L1 expression levels and the primary site. A higher proportion of patients with the primary site of the tongue or floor of the mouth had relatively low PD-L1 expression levels, whereas a higher percentage of patients with primary sites of buccal mucosa and lips had high PD-L1 expression levels. This suggests that OSCC with the same histological classification can nevertheless exhibit different PD-L1 expression levels based on its origin and the condition of the surrounding tissues.

In line with previous findings, in our study, factors showing significant differences in the survival rate analysis included tumor size, cTNM, pTNM, histopathological grade, cervical nodal metastasis, locoregional recurrence, and distant metastasis [16,17,18]. Each of these factors affect the survival rate and, simultaneously, show a correlation with PD-L1 expression. Notably, in the Kaplan–Meier curve analysis, cervical nodal metastasis and locoregional recurrence had strong effects on the survival rate of patients with OSCC. According to the cervical nodal metastasis status, the 5-year survival rate was 81.9% for patients without metastasis and 20.1% for those with metastasis, representing the largest difference in the log-rank test for each factor. Patients with different PD-L1 expression levels showed a significant difference in the survival rate. The group of patients with a high PD-L1 expression had a poorer prognosis than those with a low PD-L1 expression, with 5-year survival rates of 53.1% and 65.7%, respectively.

In addition, cervical nodal metastasis, locoregional recurrence, PD-L1 expression, and pTNM (in this order) showed a relative risk that was significantly higher than 1.0 (hazard ratio). This was in line with the log-rank test results from the survival rate analysis, indicating the relative contribution of the negative effects of each factor on the survival rate. Previous studies on the prognosis of OSCC and nodal metastasis reported that PD-L1 and PD-1 expression levels were increased by 64.9% and 61.9%, respectively, in overall OSCC, and showed a positive correlation with nodal metastasis [19,20].

To determine the role of PD-L1 in OSCC cells, we conducted an in vitro study in addition to clinical-epidemiological analyses. Cell viability, proliferation, scratch wound healing, and invasion ability were analyzed using two OSCC cancer cell lines, in which the PD-L1 expression was knocked down using PD-L1–targeted siRNA.

A decrease in cell count after PD-L1 knockdown was observed using the CCK-8 assay. The number of SAS and YD38 cells was decreased at 48 h, presumably due to the apoptosis induced by PD-L1 knockdown, suggesting an association between apoptosis and PD-L1 inhibition. Furthermore, the nuclear fragmentation assay demonstrated a significant increase in the fragmentation rate and an increase in apoptotic bodies in both SAS and YD38 cells following PD-L1 knockdown. Based on these results, we hypothesized that PD-L1 knockdown in OSCC cells induces apoptosis [21,22,23]. As expected, the protein levels of PD-L1 were significantly decreased by PD-L1 siRNA. In contrast, cleaved PARP levels were significantly increased in cells with PD-L1 knockdown. Increased levels of cleaved PARP indicated an increase in apoptosis.

Following this, an invasion assay and colony-forming assay were performed to examine the effects of apoptosis induced upon PD-L1 knockdown on cancer cells. Notably, the invasion assay showed strong effects of PD-L1 knockdown in SAS cells after 48 h of transfection. The number of cells was significantly decreased upon PD-L1 knockdown compared to the control group. This implies that the increase in apoptosis caused by PD-L1 knockdown inhibits the invasiveness and migration of OSCC cells, thereby reducing tumorigenicity [22,24,25].

Our in vitro study results showed that apoptosis increased cell death, indicating a direct or indirect association between PD-L1 protein and the apoptosis pathway in cancer cells. Ghebeh et al., in their study on breast cancer chemotherapy, observed active apoptosis similar to that seen in doxorubicin-induced apoptosis in breast cancer cells after siRNA-mediated PD-L1 knockdown, suggesting an inhibitory role of PD-L1 on apoptosis in breast cancer cells [26]. Similarly, Zhou et al. reported that PD-L1 knockdown and HIF1α significantly delayed tumor growth and metastasis [27].

The PI3K/Akt pathway plays a core regulatory role in cancer signal transduction. Our results demonstrated that PD-L1 knockdown suppressed Akt phosphorylation and the induction of Stat3 phosphorylation. In addition, SC79 pretreatment of SAS and YD38 cells followed by PD-L1 siRNA transfection induced a slight decrease in phosphorylated Stat3, suggesting that the Akt−Stat3 pathway is associated with PD-L1 expression in OSCC cells. Consistent with our findings, Abdelhamed et al. reported that inhibition of the PI3K/Akt pathway can induce compensatory activation of Stat3 [28], and that the Akt−Stat3 pathway in EGFR-activated cells can regulate PD-L1 expression [29]. In non-small cell lung cancer, the Akt−Stat3 pathway has been reported to be a promising target for immunotherapy targeting PD-L1 [29], and Choi et al. reported the association between PD-L1 and pStat3 in OSCC cells [30]. Thus, the Akt−Stat3 pathway can also be a new candidate for immunotherapy in OSCC.

Our study has several limitations. First, the sample size (81 cases) of our study is relatively small, making it difficult to draw meaningful conclusions. Initially, 541 patients with OSCC were selected and studied, but the number of OSCC patients was reduced to 81 through the five inclusion criteria. Second, additional exploration of specific signal pathways and mechanisms related to PDL1 in OSCC is required. In addition, the research of the mechanism on the reduction of the tumor forming ability induced by PDL1 knockout is needed, which is not clearly addressed in this study. This should be the focus of subsequent study on OSCC. In addition, large-scale cohort and animal model studies are needed to fully verify the results of our current research and to explain the related mechanisms underlying this phenomenon.

To summarize, the combined results of pTNM, histopathological grade, and PD-L1 expression levels obtained postoperatively can be useful for predicting the prognosis of patients and determining their subsequent treatment. Our results suggest that PD-L1 expression in tumor specimens is associated with the malignancy of OSCC and is involved in tumor growth and metastasis.

These results can be useful for the application of antibody agents that primarily target the PD-1/PD-L1 axis when PD-L1 is overexpressed, as revealed by the IHC analysis of tissues obtained after radical surgery and may contribute to the development of immunotherapy strategies for patients with OSCC.

## Figures and Tables

**Table 1:** Clinicopathological factors of all patients and χ^2^ test of the PD-L1 value status.

Parameter	*n* (%)	PD-L1 Low	PD-L1 High	*p* Value
Gender				
Male	49 (59.8%)	29	20	0.271
Female	32 (40.2%)	17	15	
Age (mean = 61.6 ± 13.6)				
≤60	35 (43.2%)	22	13	0.032 *
>60	46 (56.8%)	24	22	
Drinking +	26 (31.6%)	17	10	0.070
Smoking +	27 (32.8%)	15	11	0.881
Primary sites				
Mx.	16 (19.6%)	9	7	<0.000 **
Mn.	28 (35.8%)	14	14	
Tongue + FOM	25 (29.6%)	19	6	
Buccal mucosa + etc	12 (14.8%)	4	8	
Tumor size				
T1-T2	46 (54.9%)	30	16	<0.000 **
T3-T4	35 (45.1%)	16	19	
Clinical stage				
I + II	35 (43.2%)	27	8	<0.000 **
III + IV	46 (56.8%)	19	27	
Histopathologic grade				
Well differentiated	49 (60.5%)	28	21	0.010 *
Moderately differentiated	28 (34.6%)	17	11	
Poorly differentiated	4 (4.9%)	1	3	
Pathological stage				
I + II	32 (39.5%)	22	10	<0.000 **
III + IV	49 (60.5%)	24	25	
Cervical nodal metastasis				
No	51 (63.0%)	31	20	0.033 *
Yes	30 (36.8%)	15	15	
Locoregional recurrence				
No	56 (69.1%)	34	22	0.023 *
Yes	25 (30.9%)	12	13	
Distant metastasis				
No	73 (90.1%)	42	31	0.359
Yes	8 (9.8%)	4	4	

Mx., maxilla; Mn., mandible; FOM, floor of mouth. * *p* values are from a χ^2^ test and were statistically significant when <0.05 (two-sided). ** *p* values are from a χ^2^ test and were statistically significant when <0.01 (two-sided).

**Table 2:** Cox proportional hazards model with multivariate survival analysis for patients with OSCC.

Variable	Multivariate Survival Analysis		
	Hazard Ratio	95% CI	*p* Value
Age	0.888	0.639–1.234	0.478
Primary site	1.143	0.985–1.327	0.078
pTNM	1.419	1.159–1.737	0.001 **
Cervical nodal metastasis	3.053	2.080–4.480	<0.000 **
Locoregional recurrence	1.837	1.327–2.542	<0.000 **
PD-L1	1.573	1.123–2.203	0.008 **

pTNM, pathologic TNM stage; CI, confidence interval. ** *p* values are from a χ^2^ test and were statistically significant when <0.01 (two-sided).

## Data Availability

The datasets used and/or analyzed during the current study are available from the corresponding author upon reasonable request.

## Supplementary Materials

The following supporting information can be downloaded at: https://www.mdpi.com/article/10.3390/life12020238/s1, supplementary files S1–S4: Original western blotting figures.

## Abbreviations

NOM	Normal oral mucosa
OSCC	Oral squamous cell carcinoma
IHC	Immunohistochemistry
Mx	Maxilla
Mn	Mandible
FOM	Floor of mouth
pTNM	Pathologic TNM stage
CI	Confidence interval
SD	Standard deviation
