# Supplementary figures and images for: PD-L1 Expression Correlated with Clinicopathological Factors and Akt/Stat3 Pathway in Oral SCC
Source: Life (Basel). 2022 Feb 4;12(2):238. doi: 10.3390/life12020238 (PMC8875073; doi:10.3390/life12020238)

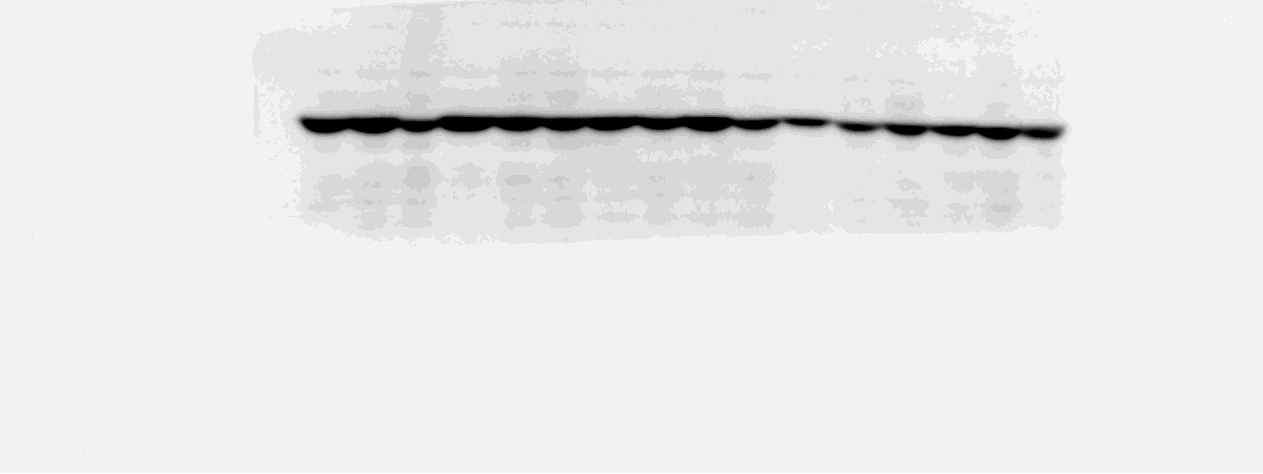

Supplement: Supplementary file 1 [file life-12-00238-s001.zip › life-1555904/supplementary file S1/beta actin.tif]

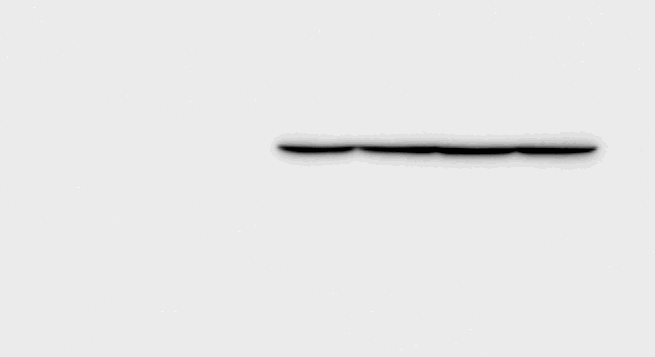

Supplement: Supplementary file 1 [file life-12-00238-s001.zip › life-1555904/supplementary file S4/SAS, YD38 - beta-actin.tif]
